# PLZF limits enhancer activity during hematopoietic progenitor aging

**DOI:** 10.1093/nar/gkz174

**Published:** 2019-03-20

**Authors:** Mathilde Poplineau, Julien Vernerey, Nadine Platet, Lia N’guyen, Léonard Hérault, Michela Esposito, Andrew J Saurin, Christel Guilouf, Atsushi Iwama, Estelle Duprez

**Affiliations:** 1 Epigenetic Factors in Normal and Malignant Hematopoiesis, Aix Marseille Université, CNRS, INSERM, Institut Paoli-Calmettes, CRCM, Marseille, France; 2 Department of Cellular and Molecular Medicine, Graduate School of Medicine, Chiba University, Chiba, Japan; 3 Gustave Roussy, Université Paris-Saclay, Inserm U1170, CNRS Villejuif, France; 4 Aix Marseille Université, CNRS, IBDM, Marseille, France; 5 Division of Stem Cell and Molecular Medicine, Center for Stem Cell Biology and Regenerative Medicine, Institute of Medical Science, University of Tokyo, Tokyo, Japan

## Abstract

PLZF (promyelocytic leukemia zinc finger) is a transcription factor acting as a global regulator of hematopoietic commitment. PLZF displays an epigenetic specificity by recruiting chromatin-modifying factors but little is known about its role in remodeling chromatin of cells committed toward a given specific hematopoietic lineage. In murine myeloid progenitors, we decipher a new role for PLZF in restraining active genes and enhancers by targeting acetylated lysine 27 of Histone H3 (H3K27ac). Functional analyses reveal that active enhancers bound by PLZF are involved in biological processes related to metabolism and associated with hematopoietic aging. Comparing the epigenome of young and old myeloid progenitors, we reveal that H3K27ac variation at active enhancers is a hallmark of hematopoietic aging. Taken together, these data suggest that PLZF, associated with active enhancers, appears to restrain their activity as an epigenetic gatekeeper of hematopoietic aging.

## INTRODUCTION

Transcription factors (TFs) play important roles during hematopoiesis, from stem cell maintenance to lineage commitment and differentiation (1,2). It is now well admitted that sequence specific TFs in concert with epigenetic factors regulate gene expression during hematopoiesis (3,4). For more than a decade, the progression of hematopoietic stem cells (HSCs) toward more differentiated cells has been thought to be accompanied by epigenetic reprograming (5,6) and it appears that the structure of chromatin is essential for hematopoietic lineage specification (7,8). However, the interdependent interactions between TFs and chromatin features during hematopoietic differentiation are still poorly understood. Studies in different systems have indicated that the chromatin of regulatory elements is in a pre-active state in stem cells and/or early progenitors before the transcriptional initiation, leading to the concept of ‘gene priming’ (9). The priming is thought to be driven by a specific class of TFs called ‘pioneer TFs’, that are able to induce the early chromatin changes during the gene activation process (10).

PLZF, also known as Zbtb16, is a master transcriptional regulator with effects on growth, self-renewal and differentiation with a well-recognized role in hematopoietic, spermatogonial, mesenchymal and neural progenitor cells (11). Within the hematopoietic tissue, PLZF is involved in the production of numerous different hematopoietic and immune cells and regulates immune responses (12,13). Its expression marks NKT-cell development (14) but also controls megakaryopoiesis (15) or invariant natural killer T cell effector functions (16). However, PLZF is not considered as a lineage-specific TF unlike lineage-instructive TFs such as PU.1 and C/EBPalpha (17–19). Indeed, PLZF inactivation in mouse models does not result in a specific blockage in the immature hematopoietic compartment (20), but induces subtle changes in HSC cell cycle progression that participate to the aging of the hematopoietic system (21). This aging-like phenotype in PLZF-mutant HSC was characterized by an increase in myeloid progenitor differentiation at the expense of the lymphoid progenitors and was correlated with the alteration of gene expression programs related to stem cell function and cell cycle (21).

PLZF has a recognized epigenetic function; it is probably through its chromatin activity that PLZF finely and precisely regulates transcriptional programs that mediate its biological functions. However, it remains unclear how PLZF causes changes in the existing epigenetic landscape (22). PLZF binds to multiple epigenetic cofactors that could be recruited and modify the chromatin landscape at the vicinity of PLZF chromatin localization (23–25). For instance, previous studies have shown that PLZF recruits HDAC complexes at targeted promoters and locally reduced histone acetylation (25,26). In addition, we previously showed that presence of PLZF at chromatin is associated with the tri-methylation of lysine 27 of histone H3 (H3K27me3) enrichment at developmental genes (24,27).

Here, we reveal a new chromatin function of PLZF in myeloid progenitors. By analyzing epigenomic landscape variations upon PLZF expression or inactivation, we discovered that PLZF inhibits acetylation of lysine 27 of histone H3 (H3K27ac) at enhancer regions that are already active. Moreover, the de-repression of the enhancer regions observed in PLZF-mutated granulocytic-monocytic progenitors (GMPs) is also observed in old GMPs. Thus, we propose that PLZF limits some aging features by restricting enhancer activity of genes involved in metabolic processes.

## MATERIALS AND METHODS

### Mice

The mouse model C57BL/6 Cd45-2 *Zbtb16^lu/lu^* was previously described in Vincent-Fabert *et al.* (21). As the *Zbtb16^lu/lu^* mutation increases the GMP compartment after regenerative stress we performed bone marrow (BM) reconstitution of *Zbtb16^lu/lu^* and WT mice. For reconstitution 2 millions of total BM were transplanted in lethally irradiated (8–8.5 Gy) C57BL/6 Cd45-1 mice. Reconstitution was monitored every 4 weeks and mice were sacrificed for GMP purification at 16 weeks post transplantation. Young (∼2 months) and aged (∼18 months) C57BL/6-Cd45.1 mice were purchased from Charles River Laboratories. B6-Cd45.1/Cd45.2 mice were bred and maintained in the CRCM mouse facility in accordance with our institutional guidelines for the use of laboratory animals and approved by the French authority (authorization number: MESR#5645).

### Purification of granulocyte monocyte progenitors (GMPs), LK and LSK

Tibias and femurs were crushed in PBS containing 3% of fetal calf serum (FCS). Red blood cells were lysed using ACK buffer (Gibco). Bone marrows were depleted in mature cells, expressing the lineage markers Cd5, B220, Cd11b, Gr-1, Ter-119 or 7-4, using the lineage cell depletion kit (Miltenyi Biotec). GMPs (Lineage^−^, Cd45^+^, C-Kit^+^, Sca-1^−^, Cd34^+^, FcγR^+^), LK (Lineage^−^, Cd45^+^, C-Kit^+^, Sca-1^−^), LSK (Lineage^−^, Cd45^+^, C-Kit^+^, Sca-1^+^) were purified using the FACS Aria III cell sorter (Beckman Dickinson). Antibodies used for GMP staining are described in supplemental materials.

### Cell culture and lentiviral transduction

The 416b murine myeloid cell line expressing the surface marker Cd34 (a generous gift from B. Göttgens) was maintained at exponential growth in RPMI 1640, 10% FCS and 1% penicillin/streptomycin. Cells were transduced with PLZF-FLAG-GFP or empty-FLAG-GFP lentiviral particles, 30 min at 2000 rpm, 32°C. After 48 h, GFP positive cells were purified on the FACS-ARIA II cell sorter (Beckman Dickinson).

### Luciferase assay

416b cells were transfected using Amaxa^®^ Cell Line Nucleofactor^®^ kit C (Lonza), U937 program, with 2 μg of *RENILLA* Luciferase control reporter and 4 μg of *CD47* enhancer-Firefly vectors. *CD47* enhancer-*FIREFLY* vector was generated from the previously described construct *CD47*-E5 (28). Luciferase activity was monitored 8 h after transfection using the Dual Luciferase Reporter Assay (Promega).

### Reverse-transcription and PCR quantitative (RT-qPCR)

Total RNA was extracted using the RNeasy Plus Micro kit (Qiagen). cDNA was synthesized with the Transcriptor High Fidelity cDNA Synthesis Kit (Roche). PCR was done using Taqman probes (Mm00607939_s1 Actb, Mm01198489_g1 Rpl6, and Mm01176868_m1 Zbtb16; Life Technologies) and Taqman Universal PCR Master Mix (Life Technologies) on the Fast 7500 Real-Time PCR system (Applied Biosystem). Relative expression levels were determined by the 2^–ΔCT^ method using *Actb* or *Rpl6* as housekeeping genes.

### Western blotting (WB)

Total proteins were extracted using the cell lysis and fractionation kit (ThermoFisher) or RIPA buffer. Proteins were dosed using the BCA methods, separated under denaturation conditions on SDS-PAGE, blotted on nitrocellulose membrane and incubated with different primary antibodies listed in supplemental experimental procedure. For Flag and Tubulinα detection, ECL prime™ (GE Healthcare) was applied. PLZF was detected using more sensitive ECL (ECL select™, GE Healthcare).

### Chromatin immunoprecipitation (ChIP)

For histone mark ChIP-seq, purified GMPs or 416b cells were fixed with 1% of formaldehyde for 8 min. Reaction was quenched by adding 2 mM of glycine. ChIP procedures are developed in supplemental experimental procedure. ChIP-seq libraries were generated using the MicroPlex Library Preparation Kit (Diagenode) following the manufacturer's instructions and analyzed on a 2100 Bioanalyzer system (Agilent) prior sequencing.

### Quantification of immunoprecipitated DNA

Quantification of immunoprecipated DNA was performed by quantitative real time PCR (qPCR). qPCR was performed using SsoAdvanced™ Universal SYBR^®^ Green Supermix (BIO-RAD) with the CFX PCR system (Bio-Rad). For enrichment quantification, Input Ct values were subtracted to ChIP Ct values and converted into bound value by 2^(–(Input Ct – ChIP Ct))^. Data are expressed as % of bound/input. Primer sequences are shown in supplemental materials.

For normalization of ChIP-qPCR, Spike-in Drosophila Chromatin and Spike-in Antibody (Active Motif) were added to the ChIP reaction as a minor fraction of the IP reaction according to the manufacturer's instructions. The immunoprecipitated Drosophila chromatin was quantified using Drosophila Positive Control Primer Set Pbgs (Active Motif), allowing the attribution of a spike-in factor to each sample, used for normalization.

### ATAC-seq

ATAC-seq was performed on 30 000 cells. Cells were washed twice with cold PBS and suspended in lysis buffer (10 mM Tris–HCl pH 7.4, 10 mM NaCl, 3 mM MgCl_2_, 0.1% IGEPAL CA-630). The same amount of cells was used for input preparation. Transposition and library preparation were done using the Nextera DNA Library Prep Kit (Illumina) according to the manufacturer's instruction. Size selection was performed using the Blue Pippin™ system.

### RNA-seq

Total RNA was extracted using the RNeasy Plus Mini kit (Qiagen). GMPs cDNA synthesis and library preparation were done by Beckman Coulter Genomics using a TruSeq Stranded Total RNA with Ribo Zero Gold kit (Illumina). 416b cDNA was synthesized using a SMART-Seq v3 Ultra Low Input RNA kit for Sequencing (Clontech). ds-cDNA was fragmented using S220 Focused ultrasonicator (Covaris) and libraries were generated using a NEBNext Ultra DNA Library Prep kit (New England Biolabs).

### Sequencing and data processing

For GMPs, ChIP-sequencing was performed with a Next-seq500 sequencer (Illumina) using a 75-nt single-end protocol, at the Paoli Calmettes Institute Sequencing Facility (IPC, Marseille) and RNA-sequencing with a Next-seq500 sequencer (Illumina) using a 100-nt paired-end protocol, at Beckman Coulter Genomics. For 416b cells, ChIP and RNA sequencing was performed with a HiSeq1500 sequencer (Illumina) using a 61-nt single-end protocol at Chiba University, Japan. Computational analyses are developed in supplemental experimental procedure. Profiles of histone ChIPseq and ATAC-seq signals were obtained by processing normalized bigwig files through the deepTools suite (v2.2.4) (29) (computeMatrix, plotProfile). For PLZF ChIP-seq, genomic distribution and annotation of PLZF peaks in 416b cell line were determined using HOMER software (v4.7.2; annotatePeaks module) (30). All sequencing data were visualized using the Integrative Genomics Viewer (IGV v2.3.92) (31).

Biological functions associated with PLZF-ChIP peaks were determined using Genomic Regions Enrichment of Annotations Tool (GREAT v3.0.0) (32). The same tool was also used for assigning enhancers to the closest genes. The Gene Ontology (GO) biological processes associated with candidate genes were determined using g:Profiler tool with a *P*-value <0.05 and by taking into account a background. For enhancers named ‘K27ac de novo’, ‘K27ac up’, ‘K27ac unchanged’ (Supplemental Figure S4) the background used was all active enhancer-associated genes contained in the 416b PLZF-Flag and 416b Empty vector conditions. For enhancers named ‘common’ (Figure 5E), the background used was all active enhancer-associated genes contained in WT, *Zbtb16^lu/lu^*, Old and Young GMP conditions.

### Statistics

For two group analyses, we first check the normal distribution of each group (Shapiro-Wilk test) and then performed Welch's *t*-test. Density plot profile *P*-values were obtained by performing paired Welch's *t*-test on computeMatrix averaged signal matrices. Concerning Venn diagram analysis, overlap significances were computed by hypergeometric tests (phyper function, lower.tail = false). Concerning ChIP-qPCR and Luciferase assay, Mann–Whitney test was used.

## RESULTS

### PLZF restricts active epigenetic marks in myeloid cells

**Figure 1.**
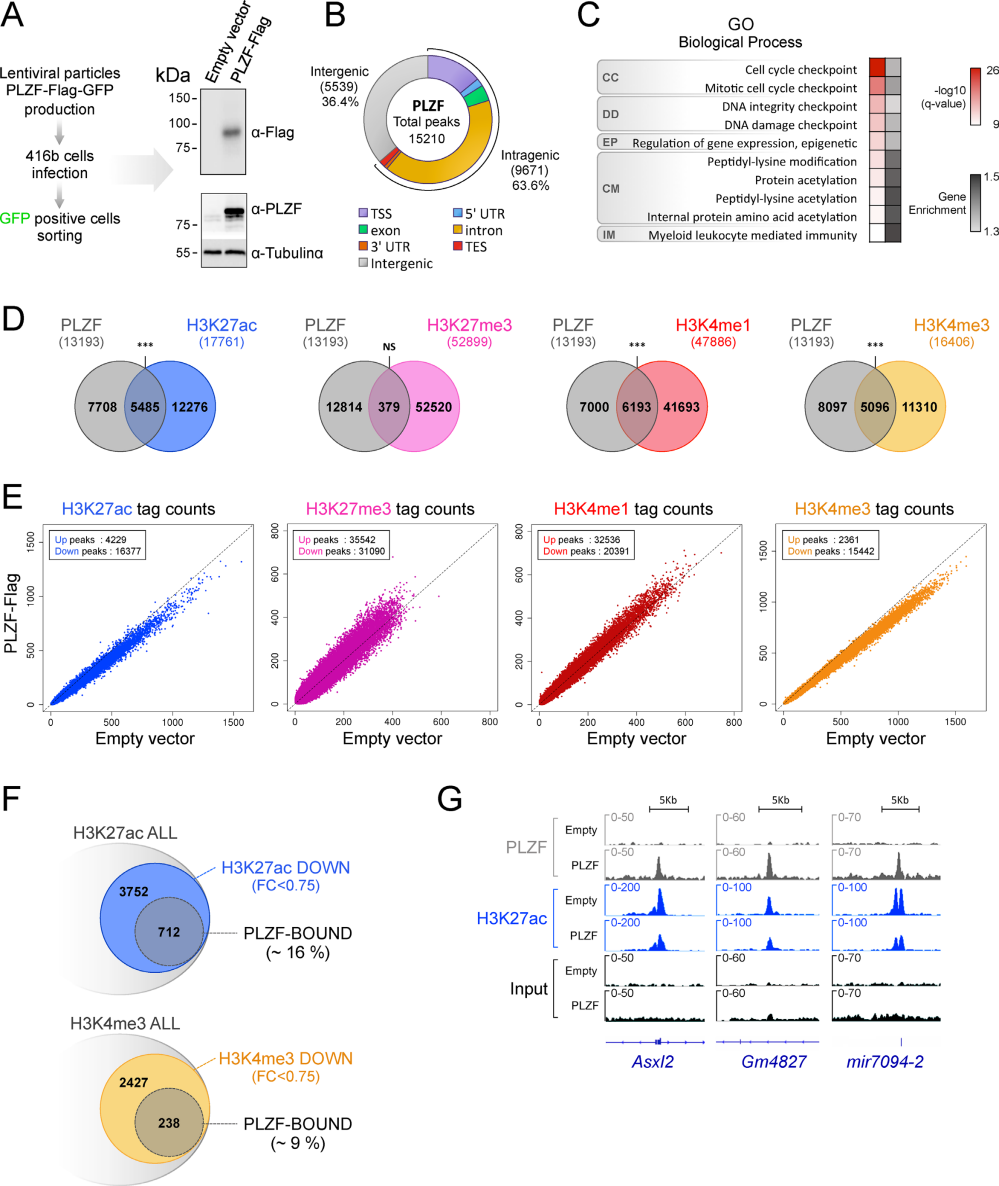
PLZF restricts active epigenetic marks in myeloid cells. (**A**) Experimental protocol design for engineering PLZF-Flag 416b cell line and immunoblot showing PLZF expression (using anti-Flag or anti-PLZF antibodies). Alpha-Tubulin was used as loading control. (**B**) Genomic distribution of PLZF obtained from PLZF-Flag transduced 416b cell line (TSS: transcription start site defined from -1kb to +100bp; TES: transcription ending site defined from –100 bp to +1 kb; UTR: untranslated region. (**C**) Gene Ontology (GO) analysis using GREAT tools of PLZF-peaks nearby genes (CC: cell cycle; DD: DNA damage; EP: epigenetics; CM: chemical modifications; IM: immunology). Gray scale values illustrate gene enrichment (i.e. number of genes observed/expected) for each GO term. (**D**) Venn diagrams showing the overlap between PLZF and histone marks.***P < 0.001 (hypergeometric test); NS not significant. (**E**) Scatter plots showing histone mark profiles in PLZF-Flag versus Empty vector conditions. (**F**) Venn diagrams showing the percentage of H3K27ac (upper) and H3K4me3 (lower) peaks modulated and/or bound by PLZF. (**G**) Integrative Genomic Viewer (IGV) screenshots of PLZF and H3K27ac in PLZF-Flag (PLZF) and Empty vector conditions (Empty).

To better understand PLZF activity in mouse myeloid cells, we generated an over PLZF-expressing mouse model by transducing the murine myeloid 416b cell line with lentivirus containing PLZF-Flag. The 416b cell line is a cellular model for Cd34-positive progenitors that express, albeit at low level, PLZF (Figure 1A).

This stable cell line was first used to profile PLZF genomic occupancy using Flag antibody. In PLZF-Flag 416b cells, PLZF binds mostly intragenic regions (63.6%) with preference for intronic sequences and TSS regions, in accordance with previously reported genomic localization in the KG1 human myeloid cells (CD34 positive cell line) (27) (Figure 1B). Presence of PLZF on its previously described targeted genomic loci was confirmed by Integrative Genomics Viewer (IGV) visualization (Supplemental Figure S1A) and peak calling analysis. In addition, 38% of the over-expressed PLZF binding sites in 416b were overlapping with PLZF endogenous binding in KG1 cells; this finding strengthens the pertinence of using the 416b line for studying PLZF chromatin activity (Supplemental Figure S1B). In line with our previous study (27), genes bound by PLZF-Flag in the myeloid progenitor cell line (SupplementaryTable S1) were enriched for Gene Ontology (GO) terms relating to cell cycle, DNA integrity and immunity. Remarkably, genes involved in protein acetylation-related GO terms were also enriched within the genes targeted by PLZF (Figure 1C), underlying a potential effect of PLZF on histone lysine acetylation. To assess the epigenetic landscape of this cell line, we performed H3K27ac, H3K4me3, H3K4me1 and H3K27me3 ChIP-seq. Our analyses show that in PLZF-Flag 416b, PLZF was significantly associated with transcriptionally active histone marks. We found that more than 41% of PLZF peaks overlapped with H3K27ac and H3K4me1, 38.6% with H3K4me3. Overlap of PLZF peaks with the transcriptionally repressive histone mark H3K27me3 was low (2.9%) and not significant (Figure 1D).

Next, we analyzed the effect of PLZF overexpression on chromatin state. We performed differential analyses on histone signal in 416b in the presence (PLZF-Flag) or absence (empty vector) of PLZF. Upon PLZF expression, H3K27ac and H3K4me3 ChIP-seq signals decrease, while H3K27me3 and H3K4me1 signals slightly increase (Figure 1E and supplemental Figure S1C) suggesting that the main chromatin effect of PLZF is to restrain already active genes through changes to the landscape of histone epigenetic marks. We found that there are twice as many downregulated H3K27ac sites than downregulated H3K4me3 bound by PLZF (Figure 1F), suggesting that PLZF preferentially targets H3K27ac. Decrease in H3K27ac level at PLZF-targeted genes was confirmed by Integrative Genomics Viewer (IGV) vizualisation (Figure 1G). Altogether, these data suggest that PLZF has a direct effect on histone acetylation. When focusing on acetylation process genes (Figure 1C), analysis of RNAseq data obtained from PLZF-Flag and Empty vector conditions showed that PLZF overexpression modulates mRNA level of genes directly (e.g: *Hdac1* or *Kat2b*) or indirectly (e.g, *Chd5*) involved in acetylation machinery (Supplemental Figure S1D). This suggests that, in addition to its direct effect on acetylation, PLZF may indirectly regulate histone acetylation.

### Overexpression of PLZF modulates H3K27ac at enhancer regions in myeloid cells

**Figure 2.**
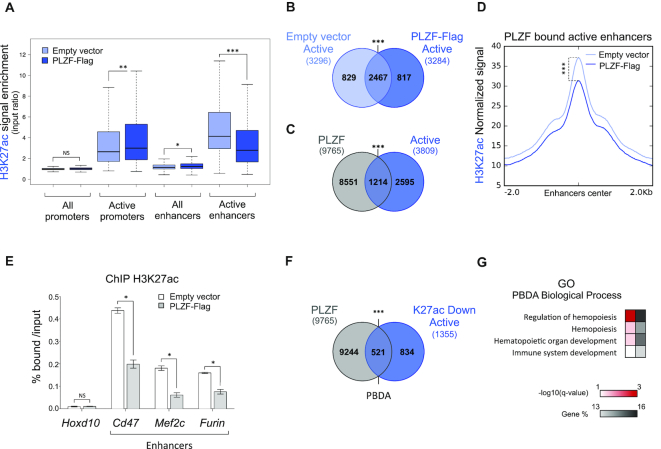
Ectopic expression of PLZF modulates H3K27ac at enhancer regions in myeloid cells. (**A**) Box plots showing H3K27ac enrichment (% H3K27ac bound/Input) in Empty vector and PLZF-Flag conditions at promoter, enhancer and active enhancer regions. *P < 0.05, **P < 0.01, ***P < 0.001 (Welch's *t*.test); NS not significant. (**B**) Venn diagrams showing the overlap of active enhancers (Active) between Empty vector and PLZF-Flag conditions. ***P < 0.001 (hypergeometric test). (**C**) Venn diagrams showing the overlap between PLZF peaks and active enhancers (Active) in PLZF-Flag condition. ***P < 0.001 (hypergeometric test). (**D**) Density plot profiles of H3K27ac normalized signal at PLZF-bound active enhancers in PLZF-Flag and Empty vector conditions. ***P < 0.001 (paired Welch's *t* test). (**E**) Spike-in ChIP-qPCR validation of H3K27ac variation at *Cd47, Furin* and *Mef2c* enhancers. *Hoxd10* is used as a negative control. Percentage of bound DNA over input are shown as a mean ±SD of two independent experiments (*n* = 3, for each experiment). *P < 0.05 (Mann–Whitney test), NS not significant. (**F**) Venn diagrams showing the overlap between PLZF peaks and active enhancers with decreased H3K27ac level (K27ac Down Active) in PLZF-Flag condition. PBDA: PLZF-Bound Down Active. In figures B and E, ***P < 0.001 (hypergeometric test); NS not significant. (**G**) Gene Ontology (GO) enrichment analysis on PBDA (intersect Figure E) using g:Profiler. Red scale indicates the *P*-value (–log_10_) and grey scale represents gene % (i.e. % of genes observed/total number of genes within each GO term).

H3K27ac is an activating mark found at promoters and marks active enhancer regions (33). Thus, we investigated whether the decrease in H3K27ac induced by PLZF-Flag was preferentially observed at one of these genomic regions. Since enhancer activity is cell-type specific and context sensitive, we determined enhancer regulatory regions using our 416b cell-specific ChIP-seq data. Promoter regions in 416b cells were defined as previously performed on human cells (27) but using the murine (mm10) genome. Enhancer regions were defined by the presence of H3K4me1 and absence of H3K4me3 and their activity was defined depending on the presence of additional histone marks (H3K27ac and H3K27me3) (Supplemental Figure S2A). We observed that PLZF overexpression induced H3K27ac changes in active promoters and active enhancers with a highly significant decrease at active enhancers (Figure 2A, Supplemental Figure S2B), suggesting that PLZF would modify active enhancer activity in 416b cells. Then, we analyzed the overlap of active enhancers from the 416b without or with PLZF-Flag. Overlap of active enhancers from the two conditions was important (Figure 2B), suggesting that PLZF does not change enhancer status (from active to inactive) but modulates their activity. To further investigate whether PLZF is directly regulating enhancer activity, we analyzed PLZF genomic localization according to active enhancers in PLZF-Flag 416b and KG1 cells that express comparable level of PLZF (Supplemental Figure S2C). We observed that PLZF-Flag binds 40% of the active enhancers in PLZF-Flag 416b cells (Figure 2C) and that endogenous PLZF binds >50% of active enhancers in KG1 cells (Supplemental Figure S2D). PLZF occupancy at active enhancers in KG1 cells was confirmed by IGV vizualisation (Supplemental Figure S2E and F). These comparable findings highlight the affinity of PLZF for active enhancer regions. In 416b cells, enhancers were diminished in their H3K27ac level upon PLZF expression (Figure 2D). H3K27ac variations at PLZF bound active enhancers were validated by spike-in normalized ChIP-qPCR (Figure 2E). Next, we looked for the proportion of active enhancer regions that exhibit a decrease in H3K27ac (K27ac Down Active) upon PLZF expression and are bound by PLZF (PBDA, PLZF Bound Down Active, Supplementary Table S2); we showed that more than 38% of K27ac Down Active enhancers were directly bound by PLZF (Figure 2F). GO analysis using the g:Profiler database confirmed that PBDA were enriched for genes associated with hematopoiesis (Figure 2G). Altogether, these results suggest that PLZF directly limits H3K27ac at enhancer regions controlling genes related to haematopoiesis.

### PLZF binding restrains enhancer activity

**Figure 3.**
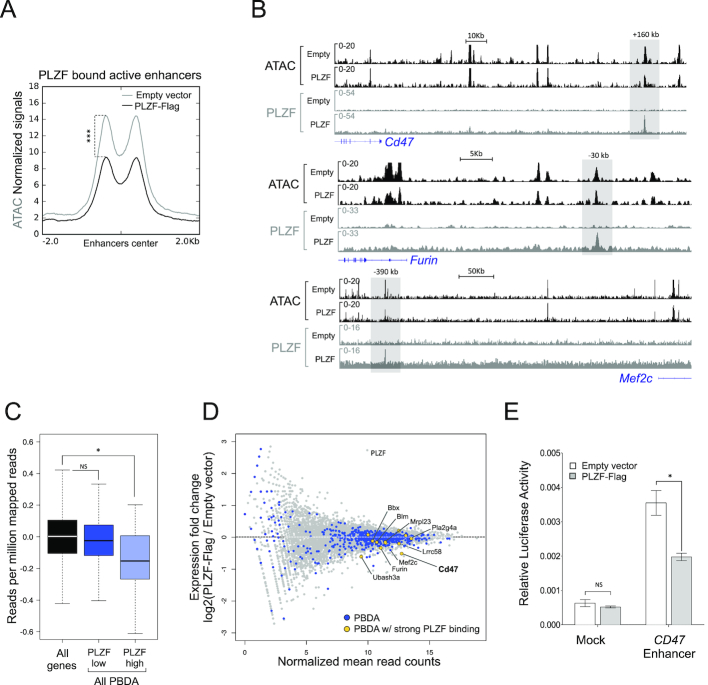
PLZF binding restrains enhancer activity. (**A**) Density plot profiles illustrating ATAC-seq normalized signal on PLZF-bound active enhancers in absence (Empty vector) or presence of PLZF (PLZF-Flag). ***P < 0.001 (paired Welch's *t* test). (**B**) Representative Integrative Genomics Viewer (IGV) tracks of ATAC-seq, PLZF, signals at *Cd47, Furin* and *Mef2c* enhancers in PLZF-Flag versus Empty vector conditions. The grey box underlines the enhancers. (**C**) Box plots showing gene expression in PLZF-Flag condition of all genes and genes associated with PBDA with low (PLZF low) or strong PLZF (PLZF high) signals. PBDA: PLZF-Bound Down Active. *P < 0.05 (Welch's *t* test), NS not significant. (**D**) MA plot showing gene expression (RNA-seq) fold changes in PLZF-Flag *versus* Empty vector conditions (grey dots). Blue dots represent genes associated with PBDA. Yellow dots represent genes associated with PBDA with strong PLZF binding. (**E**) Luciferase assay performed in 416b cells in absence (Empty vector) or presence of PLZF (PLZF-Flag) with control-LUC (Mock) or with *CD47*-enhancer-LUC (*CD47* Enhancer) reporters. Luciferase activity (FIREFLY: FF) was measured 8 h after transfection. FF values are normalized to RENILLA and expressed as a mean ± SD of three independent experiments. *P < 0.05 (Mann–Whitney test), NS not significant.

In order to evaluate the consequence of PLZF chromatin binding at enhancer regions, we measured chromatin accessibility by ATAC-seq and gene expression levels by RNA-seq in the absence and upon PLZF expression. First, ATAC-seq experiments showed that PLZF globally reduces chromatin accessibility (Supplemental Figures S3A and B). By analyzing PLZF-bound enhancer regions we revealed that when PLZF was expressed, these enhancer regions were reduced in their chromatin accessibility (Figure 3A). Decrease in ATAC-seq signal at some PLZF-bound enhancers was confirmed by IGV vizualisation (Figure 3B). These results suggest that PLZF binding at enhancers decreases their accessibility. Since enhancers can target genes located in their vicinity (34), we analyzed by RNA-seq gene expression according to the presence of PLZF in the associated enhancers. PBDA associated genes were separated into two groups depending on their PLZF ChIP-seq signal (low or high) and expression status of the two groups were compared to all genes. Results show that strong PLZF binding at enhancers (PLZF high) was associated with a significant lower gene expression in comparison to global expression (Figure 3C). When we compared RNA-seq counts from 416b cells with and without PLZF, we found that genes associated with PBDA displayed increased or reduced expression upon PLZF expression. However, genes associated with a strong PLZF binding were consistently down-regulated (Figure 3D). This suggests a direct effect of PLZF at enhancer region that is preferentially a restrictive effect on gene expression.

Finally, to recapitulate PLZF enhancer activity, we focused on one enhancer region bound by PLZF that regulates the CD47 gene (see Figure 3B). CD47 is involved in inflammatory response (35) and is recognized as an immune checkpoint for tumor evasion (36). We performed a Luciferase reporter assay using the previously described CD47 human enhancer (28) that we revealed to be bound by PLZF (extracted from Koubi *et al.*, data). Luciferase activity monitoring showed that PLZF repressed the reporter activity under control of the CD47 enhancer (Figure 3E).

Altogether these data suggest that PLZF acts directly on enhancer regions to decrease their accessibility and activity.

### PLZF mutation increases H3K27ac at enhancers targeting genes related to metabolism

**Figure 4.**
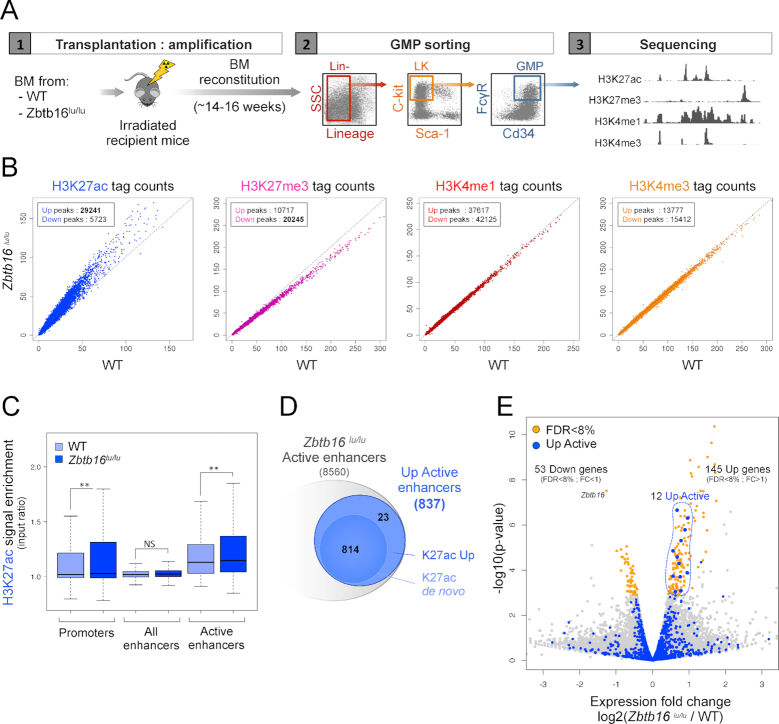
PLZF-mutation induced H3K27ac at enhancer regions in GMPs. (**A**) Experimental design used for generating histone mark ChIP-seq in wild type (WT) and PLZF mutant (*Zbtb16^lu/lu^*) Granulocyte-Monocyte Progenitors (GMPs). Lin-: Lineage negative, LK: Lin-, c-kit^+^ and Sca-1^−^. (**B**) Scatter plots showing H3K27ac, H3K27me3, H3K4me1 and H3K4me3 signal in *Zbtb16^lu/lu^ versus* WT GMP. (**C**) Box plots showing H3K27ac enrichment (% H3K27ac bound/input) in WT and *Zbtb16^lu/lu^* GMPs at promoter, enhancer and active enhancer regions. **P < 0.01 (Welch's *t* test), NS not significant. (**D**) Venn diagrams showing the numbers of active enhancers in the *Zbtb16^lu/lu^* GMPs. K27ac Up: upregulated compared to the WT condition, K27ac *de novo*: not detected in the WT condition. (**E**) Volcano plot representing differential gene expression (RNA-seq) in *Zbtb16^lu/lu^*compared to WT. Yellow dots represent genes significantly modulated (false discovery rate (FDR) < 8%, fold change (FC) > 1 or < 1). Up Active enhancers are highlighted in blue. The 12 Up-Active associated genes with FDR <8% that appear in blue are circled in blue.

PLZF is expressed in the immature myeloid compartment of mouse bone marrow (BM) with higher expression in Granulocyte-Monocyte Progenitor (GMP) compartment (Supplemental Figure S4A). Besides, we previously showed that PLZF inactivation increases the GMP compartment after regenerative stress in the *Zbtb16^lu/lu^* mouse model (21). To question whether H3K27ac regulation by PLZF is linked to the GMP phenotype, we compared the chromatin landscape of *Zbtb16^lu/lu^* to WT GMPs after BM transplantation (Figure 4A). We profiled H3K27ac, H3K4me3, H3K4me1 and H3K27me3 distribution in WT and *Zbtb16^lu/lu^* GMPs. In accordance to our gain-of-function PLZF-Flag 416b cell line model, we showed that PLZF inactivation *in vivo* increased H3K27ac at the genomic level while H3K27me3 slightly significantly decreased (Supplemental Figure S4B) and the other epigenetic marks tested were modestly affected (Figure 4B). H3K27ac increase upon PLZF-mutation occurred both at promoter and enhancer regions (Figure 4C). In accordance with our overexpression model, we observe a stronger H3K27ac variation in mutant compared to WT when only active enhancers were considered (Figure 4C, Supplemental Figure S4C). In addition, a significant overlap was found between enhancers modified upon PLZF overexpression (Down Active) and upon PLZF mutation (Up Active) emphasizing the fact that PLZF globally restricts enhancer activity (Supplemental Figure S4D). Next, we investigated whether PLZF mutation favored new H3K27ac enriched putative enhancers (‘*de novo* active enhancers’) or was triggering already active enhancers. We showed that PLZF mutation increased H3K27ac at enhancer regions in both situation, when H3K27ac signal was detected in WT GMP (K27Ac Up: ‘already’ active enhancers) but also when H3K27ac was not detected by our peak calling (K27Ac *de novo*) (Figure 4D, Supplemental Figures S4E and F). When considering all active enhancers identified in Mutant and WT GMPs (background, see Materials and Methods), ‘*de novo*’ and ‘already’ active enhancers modulated upon PLZF mutation exhibited a specific gene signature compared to unmodulated enhancers highlighting the specificity of PLZF on enhancer regions (Supplemental Figure S4G, Table S3). Indeed modulated enhancers were enriched for genes involved in response to stimulus and phosphorylation processes (Supplemental Figure S4G) whereas no significant gene signature was found for unaffected enhancers. To analyse whether changes in the epigenetic landscape would affect gene expression, we performed RNA-seq and compared the transcriptome of *Zbtb16^lu/lu^* to WT GMPs after BM transplantation. We showed that PLZF inactivation slightly affected gene expression (53 downregulated and 145 upregulated) and that significantly affected (FDR<8%) Up-active enhancer associated genes were found up regulated in absence of PLZF (Figure 4E, Supplementary Table S4). In addition, we analysed the expression of genes known to be involved in acetylation/deacetylation processes in the *Zbtb16^lu/lu^* and WT GMPs. We found that PLZF inactivation significantly affected three genes encoding indirect partners of acetylation machinery (Supplemental Figure S4G) highlighting an indirect role for PLZF in chromatin acetylation.

Altogether, these results show that PLZF inactivation results in an increase in H3K27ac at enhancer regions and suggest its important role for chromatin regulation in myeloid progenitors.

### Aging of GMPs is marked by an accumulation of H3K27ac at enhancer regions

**Figure 5.**
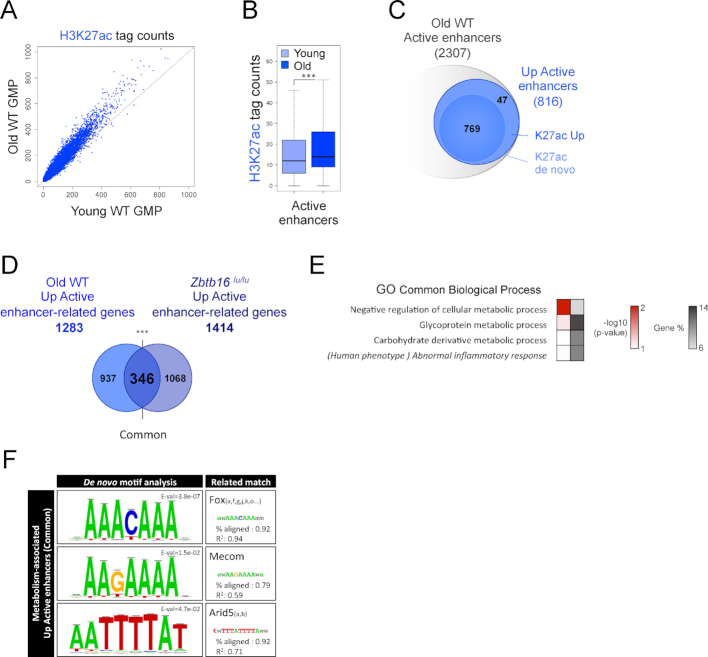
Aging of GMPs is marked by an accumulation of H3K27ac at enhancer regions. (**A**) Scatter plots showing H3K27ac profiles in Old versus Young GMPs. (**B**) Box plot showing H3K27ac level at active enhancers in Old *versus* Young GMPs. ***P < 0.001 (Welch's *t* test). (**C**) Venn diagrams showing the numbers of active enhancers in Old WT GMPs. K27ac Up: upregulated compared to the Young condition, K27ac *de novo*: not detected in the Young condition. (**D**) Venn diagram showing the overlap (common) between genes associated with active enhancers upregulated during aging (Old Up Active associated genes) and genes associated with active enhancers upregulated in *Zbtb16^lu/lu^* GMPs (*Zbtb16^lu/lu^* Up Active associated genes) ***P < 0.001 (hypergeometric test). (**E**) Gene Ontology (GO) analysis and associated human phenotype of ‘common’ enhancers that change in aged and *Zbtb16^lu/lu^* conditions (intersect Figure D) using g:Profiler. Red scale indicates the *P*-value (–log_10_) and grey scale represents gene % (i.e. % of genes observed /total number of genes within each GO/KEGG term). (**F**) *De novo* binding motif analysis at ‘common’ enhancers associated with metabolic processes.

We previously showed that PLZF limits some of the HSC aging features (21). Thus, we asked whether the PLZF specificity on enhancers was linked to hematopoietic aging. For this purpose, we investigated H3K27ac modulation in GMP compartment of aged mice. Comparison of H3K27ac level in Old and Young WT GMPs revealed a global H3K27ac increase in Old GMPs (Figure 5A). Comparable to what we observed in *Zbtb16^lu/lu^* GMPs, this increase was significant at active enhancer regions (Figure 5B). Among the 2307 putative active enhancers found in old GMPs, 816 gained H3K27ac upon aging. This gain of H3K27ac was mostly observed in ‘*de novo*’ active enhancers (Figure 5C). Interestingly, among the 1283 genes associated with these enhancers that gain H3K27ac upon aging (Old WT Up Active), 27% (346 genes, ‘common’) were also affected by PLZF deletion (*Zbtb16^lu/l^*^u^ Up Active) (Figure 5D, Supplementary Table S5). Remarkably, 86% of these conjointly modulated enhancers were potentially bound by PLZF (Supplemental Figure S5A). When considering all active enhancers identified in Young, Old, WT and Mutant GMPs as background enhancer set (see materials and methods), these conjointly regulated enhancer regions (common) were enriched in genes associated with metabolism (Figure 5E). They were also related to abnormal inflammatory response when extending the analysis to associated-human phenotype (Figure 5E). Interestingly, considering the same background enhancer set as above, no significant biological processes were found for enhancers modulated exclusively by aging (937 genes) or by PLZF deletion (1068 genes). Next, we asked whether these conjointly modulated enhancers were enriched for any specific binding motifs (Figure 5F) in comparison to enhancers unaffected by PLZF inactivation (Supplemental Figure S5B). We found that these ‘common’ enhancers were enriched for Mecom and Arid5(a,b) binding motifs. This suggests that the regulation of these ‘common’ enhancers is under the control of specific transcriptional modulators and may involve a regulation cascade due to the fact that *Arid5a* expression is also affected by PLZF inactivation (Supplemental Figure S4H). As enhancers modulated upon aging appeared to be under the control of PLZF, we monitored by qPCR the expression of PLZF in Young and Old GMPs. We showed that Young and Old GMPs exhibited similar *Plzf* expression levels (Supplemental Figure S5C), suggesting that aging may alter PLZF chromatin activity rather than its expression level.

Altogether, these results show that increase in H3K27ac level at enhancer regions controlling metabolism-related genes may be a hallmark of hematopoietic progenitor aging that is controlled by PLZF activity.

## DISCUSSION

PLZF is a transcription factor involved in multiple facets of cell biology. Here we reveal a new molecular function of PLZF in limiting H3K27ac at enhancer regions that may restrict their activity. H3K27ac is a well-defined marker of active enhancers (33), and it is essential for their activation (37). Thus, by showing that PLZF controls H3K27ac level in murine progenitor cells, we highlight a novel PLZF chromatin activity that is merely to restrict active enhancers. Gain of function experiments support a direct effect of PLZF on H3K27ac and enhancer activities. This goes in line with previous studies showing that PLZF recruits HDAC complexes at targeted promoters and locally reduced histone acetylation (25,26,38). However, the global increase of H3K27ac, not restricted at PLZF binding sites, that is observed in PLZF-mutated myeloid progenitors also suggests that PLZF has an effect on acetyl regulation beyond its direct chromatin activity. This close relation between PLZF and acetylation is not only supported by our ChIP-seq experiments that revealed enrichment of PLZF at genes involved in protein acetylation but also by our RNA-seq data that showed modulation of the expression of some genes involved in the acetylation machinery and by the fact that PLZF transcriptional activity is itself modified by acetylation (39,40).

Due to its affinity with Polycomb group proteins (23,24), PLZF was first described as a transcriptional repressor. The repressive chromatin activity of PLZF has been challenged previously by genomic data analyses showing the presence of PLZF on regulatory elements of active genes (12,27,41). Here again, analyzing murine myeloid progenitors, we found that PLZF is mainly present at already active chromatin. However, by modulating its expression in myeloid progenitors (overexpression in 416b cells and mutation in GMPs), we demonstrate that the presence of PLZF reduces the H3K27ac active mark and that this reduction was marked at active enhancers emphasizing its repressive activity. This suggests that PLZF limits enhancer activity through acting as a ‘brake’ on these regulatory elements. This notion of ‘brake’ to ensure appropriate enhancer activity was previously proposed for RACK7/KDM5C complex that controls enhancer over-activation by modulating histone methylation (42).

This ‘brake’ function on enhancer activity fits well with PLZF hematopoietic function: PLZF has been described to modify chromatin to restrain the production of pro-inflammatory cytokines in case of a bacterial infection in bone marrow-derived macrophages (38). Interestingly, this restriction involves acetylation of PLZF itself that promotes the assembly of a repressor complex incorporating HDAC3 and the NF-κB p50 subunit that limits the NF-κB signaling (40).

It is also striking to notice that the strong gain of H3K27ac at enhancer regions in PLZF-mutant GMPs has little repercussion at the transcriptional level. Differential gene expression analyses revealed that only 12 H3K27ac-UP associated genes are more expressed in the PLZF mutant condition. This apparent discrepancy between chromatin structure and gene expression is not without precedent (43). Chromatin modifications may precede, or prime chromatin for, transcriptional changes. It is also reported that chromatin structure identifies hematopoietic cell population better than gene expression profile (44). This suggests that the absence of PLZF would prime chromatin to be able to respond to cell stimuli.

In line with this idea, we highlighted a clear increase in H3K27ac, marked at enhancer regions in aged myeloid progenitors, comparable to what we observed in PLZF-mutant progenitors. There is a growing body of evidence demonstrating that epigenetic changes are central to age-associated tissue decline (45). The main common aging-chromatin feature is a global loss of closed-chromatin, characterized by a decrease in H3K9me3 (46,47), a gain in H3K4me3 specifically at promoters of self-renewing genes and a global DNA hypomethylation (48). While histone acetylation, *via* the study of two deacetylases Sirt1 and Sirt3, was previously shown to play a role under stress conditions in aging mouse HSC (49,50), the direct assessment of H3K27ac in hematopoietic cell aging has not before been addressed. Here, we found that H3K27ac is a hallmark of aged GMPs and observe a loose chromatin structure at genes involved in metabolic processes while aging. Interestingly, these enhancers were marked by specific binding motifs for Mecom and Arid5, which were previously described as key partners of chromatin acetylation machinery (51,52). Besides, metabolic deregulation was reported in several aging models (21,53). Interestingly, aged-related dysfunction of metabolism could in turn affect epigenetics such as deregulation of NAD+ production upon aging that leads to enzymatic deficiency of the NAD-dependent histone deacetylase Sirt1 (54).

PLZF expression does not decline during aging but its activity may change. Recent studies have highlighted that epigenetic cofactors having post-translational modification properties could also modify PLZF itself and influence its transcriptional competence. PLZF transcriptional repression required the acetyltransferase activity of P300 (55). More recently, PLZF was shown to interact with the histone methyltransferase EZH2, which could methylate PLZF affecting its stability and its transcriptional activity (27,56). In addition, HDAC7, a key factor of the innate effector programming of iNKT cells, changes PLZF activity underlying the tight connection between specific transcription factor activity and post-translational modification of the epigenetic machinery (57).

In conclusion, we have elucidated a novel function for PLZF, a known epigenetic regulator, in restricting enhancer acetylation of genes involved in hematopoietic aging.

## DATA AVAILABILITY

ChIP-seq data and RNAseq data (FastQ and Bigwig files) are deposited in the NCBI Gene Expression Omnibus (GEO; http//www.ncbi.nih.gov/geo/) under the accession number GSE124190.

## Supplementary Material

Supplementary Data

## References

[1] Katsumura K.R., Bresnick E.H. The GATA factor revolution in hematology. Blood. 2017; 129:2092–2102. 28179282 10.1182/blood-2016-09-687871 PMC5391619

[2] Wilkinson A.C., Gottgens B. Transcriptional regulation of haematopoietic stem cells. Adv. Exp. Med. Biol. 2013; 786:187–212. 23696358 10.1007/978-94-007-6621-1_11

[3] Kosan C., Godmann M. Genetic and epigenetic mechanisms that maintain hematopoietic stem cell function. Stem Cells Int. 2016; 2016:5178965. 26798358 10.1155/2016/5178965 PMC4699043

[4] Sashida G., Iwama A. Epigenetic regulation of hematopoiesis. Int. J. Hematol. 2012; 96:405–412. 23054647 10.1007/s12185-012-1183-x

[5] Attema J.L., Papathanasiou P., Forsberg E.C., Xu J., Smale S.T., Weissman I.L. Epigenetic characterization of hematopoietic stem cell differentiation using miniChIP and bisulfite sequencing analysis. Proc. Natl. Acad. Sci. U.S.A. 2007; 104:12371–12376. 17640913 10.1073/pnas.0704468104 PMC1924790

[6] Cui K., Zang C., Roh T.Y., Schones D.E., Childs R.W., Peng W., Zhao K. Chromatin signatures in multipotent human hematopoietic stem cells indicate the fate of bivalent genes during differentiation. Cell Stem Cell. 2009; 4:80–93. 19128795 10.1016/j.stem.2008.11.011 PMC2785912

[7] Lara-Astiaso D., Weiner A., Lorenzo-Vivas E., Zaretsky I., Jaitin D.A., David E., Keren-Shaul H., Mildner A., Winter D., Jung S. et al. Immunogenetics. Chromatin state dynamics during blood formation. Science. 2014; 345:943–949. 25103404 10.1126/science.1256271 PMC4412442

[8] Petruk S., Mariani S.A., De Dominici M., Porazzi P., Minieri V., Cai J., Iacovitti L., Flomenberg N., Calabretta B., Mazo A. Structure of nascent chromatin is essential for hematopoietic lineage specification. Cell Rep. 2017; 19:295–306. 28402853 10.1016/j.celrep.2017.03.035 PMC5408750

[9] Mercer E.M., Lin Y.C., Benner C., Jhunjhunwala S., Dutkowski J., Flores M., Sigvardsson M., Ideker T., Glass C.K., Murre C. Multilineage priming of enhancer repertoires precedes commitment to the B and myeloid cell lineages in hematopoietic progenitors. Immunity. 2011; 35:413–425. 21903424 10.1016/j.immuni.2011.06.013 PMC3183365

[10] Zaret K.S., Carroll J.S. Pioneer transcription factors: establishing competence for gene expression. Genes Dev. 2011; 25:2227–2241. 22056668 10.1101/gad.176826.111 PMC3219227

[11] Liu T.M., Lee E.H., Lim B., Shyh-Chang N. Concise review: Balancing stem cell Self-Renewal and differentiation with PLZF. Stem Cells. 2016; 34:277–287. 26676652 10.1002/stem.2270

[12] Doulatov S., Notta F., Rice K.L., Howell L., Zelent A., Licht J.D., Dick J.E. PLZF is a regulator of homeostatic and cytokine-induced myeloid development. Genes Dev. 2009; 23:2076–2087. 19723763 10.1101/gad.1788109 PMC2751973

[13] Ozato K. PLZF outreach: a finger in interferon's pie. Immunity. 2009; 30:757–758. 19538924 10.1016/j.immuni.2009.06.003 PMC4151458

[14] Savage A.K., Constantinides M.G., Han J., Picard D., Martin E., Li B., Lantz O., Bendelac A. The transcription factor PLZF directs the effector program of the NKT cell lineage. Immunity. 2008; 29:391–403. 18703361 10.1016/j.immuni.2008.07.011 PMC2613001

[15] Labbaye C., Spinello I., Quaranta M.T., Pelosi E., Pasquini L., Petrucci E., Biffoni M., Nuzzolo E.R., Billi M., Foa R. et al. A three-step pathway comprising PLZF/miR-146a/CXCR4 controls megakaryopoiesis. Nat. Cell Biol. 2008; 10:788–801. 18568019 10.1038/ncb1741

[16] Kovalovsky D., Uche O.U., Eladad S., Hobbs R.M., Yi W., Alonzo E., Chua K., Eidson M., Kim H.J., Im J.S. et al. The BTB-zinc finger transcriptional regulator PLZF controls the development of invariant natural killer T cell effector functions. Nat. Immunol. 2008; 9:1055–1064. 18660811 10.1038/ni.1641 PMC2662733

[17] Dahl R., Walsh J.C., Lancki D., Laslo P., Iyer S.R., Singh H., Simon M.C. Regulation of macrophage and neutrophil cell fates by the PU.1:C/EBPalpha ratio and granulocyte colony-stimulating factor. Nat. Immunol. 2003; 4:1029–1036. 12958595 10.1038/ni973

[18] Friedman A.D. C/EBPalpha induces PU.1 and interacts with AP-1 and NF-kappaB to regulate myeloid development. Blood Cells Mol. Dis. 2007; 39:340–343. 17669672 10.1016/j.bcmd.2007.06.010 PMC2083642

[19] Iwasaki H., Mizuno S., Arinobu Y., Ozawa H., Mori Y., Shigematsu H., Takatsu K., Tenen D.G., Akashi K. The order of expression of transcription factors directs hierarchical specification of hematopoietic lineages. Genes Dev. 2006; 20:3010–3021. 17079688 10.1101/gad.1493506 PMC1620021

[20] Barna M., Hawe N., Niswander L., Pandolfi P.P. Plzf regulates limb and axial skeletal patterning. Nat. Genet. 2000; 25:166–172. 10835630 10.1038/76014

[21] Vincent-Fabert C., Platet N., Vandevelde A., Poplineau M., Koubi M., Finetti P., Tiberi G., Imbert A.M., Bertucci F., Duprez E. PLZF mutation alters mouse hematopoietic stem cell function and cell cycle progression. Blood. 2016; 127:1881–1885. 26941402 10.1182/blood-2015-09-666974

[22] Suliman B.A., Xu D., Williams B.R. The promyelocytic leukemia zinc finger protein: two decades of molecular oncology. Front. Oncol. 2012; 2:74. 22822476 10.3389/fonc.2012.00074 PMC3398472

[23] Barna M., Merghoub T., Costoya J.A., Ruggero D., Branford M., Bergia A., Samori B., Pandolfi P.P. Plzf mediates transcriptional repression of HoxD gene expression through chromatin remodeling. Dev. Cell. 2002; 3:499–510. 12408802 10.1016/s1534-5807(02)00289-7

[24] Boukarabila H., Saurin A.J., Batsche E., Mossadegh N., van Lohuizen M., Otte A.P., Pradel J., Muchardt C., Sieweke M., Duprez E. The PRC1 Polycomb group complex interacts with PLZF/RARA to mediate leukemic transformation. Genes Dev. 2009; 23:1195–1206. 19451220 10.1101/gad.512009 PMC2685534

[25] David G., Alland L., Hong S.H., Wong C.W., DePinho R.A., Dejean A. Histone deacetylase associated with mSin3A mediates repression by the acute promyelocytic leukemia-associated PLZF protein. Oncogene. 1998; 16:2549–2556. 9627120 10.1038/sj.onc.1202043

[26] Lin R.J., Nagy L., Inoue S., Shao W., Miller W.H. Jr, Evans R.M. Role of the histone deacetylase complex in acute promyelocytic leukaemia. Nature. 1998; 391:811–814. 9486654 10.1038/35895

[27] Koubi M., Poplineau M., Vernerey J., N’Guyen L., Tiberi G., Garciaz S., El-Kaoutari A., Maqbool M.A., Andrau J.C., Guillouf C. et al. Regulation of the positive transcriptional effect of PLZF through a non-canonical EZH2 activity. Nucleic Acids Res. 2018; 46:3339–3350. 29425303 10.1093/nar/gky080 PMC5909434

[28] Betancur P.A., Abraham B.J., Yiu Y.Y., Willingham S.B., Khameneh F., Zarnegar M., Kuo A.H., McKenna K., Kojima Y., Leeper N.J. et al. A CD47-associated super-enhancer links pro-inflammatory signalling to CD47 upregulation in breast cancer. Nat. Commun. 2017; 8:14802. 28378740 10.1038/ncomms14802 PMC5382276

[29] Ramirez F., Ryan D.P., Gruning B., Bhardwaj V., Kilpert F., Richter A.S., Heyne S., Dundar F., Manke T. deepTools2: a next generation web server for deep-sequencing data analysis. Nucleic Acids Res. 2016; 44:W160–W165. 27079975 10.1093/nar/gkw257 PMC4987876

[30] Heinz S., Benner C., Spann N., Bertolino E., Lin Y.C., Laslo P., Cheng J.X., Murre C., Singh H., Glass C.K. Simple combinations of lineage-determining transcription factors prime cis-regulatory elements required for macrophage and B cell identities. Mol. Cell. 2010; 38:576–589. 20513432 10.1016/j.molcel.2010.05.004 PMC2898526

[31] Robinson J.T., Thorvaldsdottir H., Winckler W., Guttman M., Lander E.S., Getz G., Mesirov J.P. Integrative genomics viewer. Nat. Biotechnol. 2011; 29:24–26. 21221095 10.1038/nbt.1754 PMC3346182

[32] McLean C.Y., Bristor D., Hiller M., Clarke S.L., Schaar B.T., Lowe C.B., Wenger A.M., Bejerano G. GREAT improves functional interpretation of cis-regulatory regions. Nat. Biotechnol. 2010; 28:495–501. 20436461 10.1038/nbt.1630 PMC4840234

[33] Creyghton M.P., Cheng A.W., Welstead G.G., Kooistra T., Carey B.W., Steine E.J., Hanna J., Lodato M.A., Frampton G.M., Sharp P.A. et al. Histone H3K27ac separates active from poised enhancers and predicts developmental state. Proc. Natl. Acad. Sci. U.S.A. 2010; 107:21931–21936. 21106759 10.1073/pnas.1016071107 PMC3003124

[34] Furlong E.E.M., Levine M. Developmental enhancers and chromosome topology. Science. 2018; 361:1341–1345. 30262496 10.1126/science.aau0320 PMC6986801

[35] Su X., Johansen M., Looney M.R., Brown E.J., Matthay M.A. CD47 deficiency protects mice from lipopolysaccharide-induced acute lung injury and Escherichia coli pneumonia. J. Immunol. 2008; 180:6947–6953. 18453616 10.4049/jimmunol.180.10.6947 PMC2771449

[36] Liu X., Kwon H., Li Z., Fu Y.X. Is CD47 an innate immune checkpoint for tumor evasion. J. Hematol. Oncol. 2017; 10:12. 28077173 10.1186/s13045-016-0381-z PMC5225552

[37] Raisner R., Kharbanda S., Jin L., Jeng E., Chan E., Merchant M., Haverty P.M., Bainer R., Cheung T., Arnott D. et al. Enhancer activity requires CBP/P300 Bromodomain-Dependent histone H3K27 acetylation. Cell Rep. 2018; 24:1722–1729. 30110629 10.1016/j.celrep.2018.07.041

[38] Sadler A.J., Rossello F.J., Yu L., Deane J.A., Yuan X., Wang D., Irving A.T., Kaparakis-Liaskos M., Gantier M.P., Ying H. et al. BTB-ZF transcriptional regulator PLZF modifies chromatin to restrain inflammatory signaling programs. Proc. Natl. Acad. Sci. U.S.A. 2015; 112:1535–1540. 25605927 10.1073/pnas.1409728112 PMC4321291

[39] McConnell M.J., Durand L., Langley E., Coste-Sarguet L., Zelent A., Chomienne C., Kouzarides T., Licht J.D., Guidez F. Post transcriptional control of the epigenetic stem cell regulator PLZF by sirtuin and HDAC deacetylases. Epigenet. Chromatin. 2015; 8:38. 10.1186/s13072-015-0030-8 PMC4581162 26405459

[40] Sadler A.J., Suliman B.A., Yu L., Yuan X., Wang D., Irving A.T., Sarvestani S.T., Banerjee A., Mansell A.S., Liu J.P. et al. The acetyltransferase HAT1 moderates the NF-kappaB response by regulating the transcription factor PLZF. Nat. Commun. 2015; 6:6795. 25865065 10.1038/ncomms7795

[41] Mao A.P., Constantinides M.G., Mathew R., Zuo Z., Chen X., Weirauch M.T., Bendelac A. Multiple layers of transcriptional regulation by PLZF in NKT-cell development. Proc. Natl. Acad. Sci. U.S.A. 2016; 113:7602–7607. 27325774 10.1073/pnas.1601504113 PMC4941452

[42] Shen H., Xu W., Guo R., Rong B., Gu L., Wang Z., He C., Zheng L., Hu X., Hu Z. et al. Suppression of enhancer overactivation by a RACK7-histone demethylase complex. Cell. 2016; 165:331–342. 27058665 10.1016/j.cell.2016.02.064 PMC4826479

[43] Kilpinen H., Waszak S.M., Gschwind A.R., Raghav S.K., Witwicki R.M., Orioli A., Migliavacca E., Wiederkehr M., Gutierrez-Arcelus M., Panousis N.I. et al. Coordinated effects of sequence variation on DNA binding, chromatin structure, and transcription. Science. 2013; 342:744–747. 24136355 10.1126/science.1242463 PMC5502466

[44] Corces M.R., Buenrostro J.D., Wu B., Greenside P.G., Chan S.M., Koenig J.L., Snyder M.P., Pritchard J.K., Kundaje A., Greenleaf W.J. et al. Lineage-specific and single-cell chromatin accessibility charts human hematopoiesis and leukemia evolution. Nat. Genet. 2016; 48:1193–1203. 27526324 10.1038/ng.3646 PMC5042844

[45] de Haan G., Lazare S.S. Aging of hematopoietic stem cells. Blood. 2018; 131:479–487. 29141947 10.1182/blood-2017-06-746412

[46] Djeghloul D., Kuranda K., Kuzniak I., Barbieri D., Naguibneva I., Choisy C., Bories J.C., Dosquet C., Pla M., Vanneaux V. et al. Age-Associated decrease of the histone methyltransferase SUV39H1 in HSC perturbs heterochromatin and B lymphoid differentiation. Stem Cell Rep. 2016; 6:970–984. 10.1016/j.stemcr.2016.05.007 PMC4911502 27304919

[47] Sidler C., Woycicki R., Li D., Wang B., Kovalchuk I., Kovalchuk O. A role for SUV39H1-mediated H3K9 trimethylation in the control of genome stability and senescence in WI38 human diploid lung fibroblasts. Aging (Albany, NY). 2014; 6:545–563. 25063769 10.18632/aging.100678 PMC4153622

[48] Sun D., Luo M., Jeong M., Rodriguez B., Xia Z., Hannah R., Wang H., Le T., Faull K.F., Chen R. et al. Epigenomic profiling of young and aged HSCs reveals concerted changes during aging that reinforce self-renewal. Cell Stem Cell. 2014; 14:673–688. 24792119 10.1016/j.stem.2014.03.002 PMC4070311

[49] Brown K., Xie S., Qiu X., Mohrin M., Shin J., Liu Y., Zhang D., Scadden D.T., Chen D. SIRT3 reverses aging-associated degeneration. Cell Rep. 2013; 3:319–327. 23375372 10.1016/j.celrep.2013.01.005 PMC3582834

[50] Singh S.K., Williams C.A., Klarmann K., Burkett S.S., Keller J.R., Oberdoerffer P. Sirt1 ablation promotes stress-induced loss of epigenetic and genomic hematopoietic stem and progenitor cell maintenance. J. Exp. Med. 2013; 210:987–1001. 23630229 10.1084/jem.20121608 PMC3646499

[51] Amano K., Hata K., Muramatsu S., Wakabayashi M., Takigawa Y., Ono K., Nakanishi M., Takashima R., Kogo M., Matsuda A. et al. Arid5a cooperates with Sox9 to stimulate chondrocyte-specific transcription. Mol. Biol. Cell. 2011; 22:1300–1311. 21346191 10.1091/mbc.E10-07-0566 PMC3078073

[52] Ivanochko D., Halabelian L., Henderson E., Savitsky P., Jain H., Marcon E., Duan S., Hutchinson A., Seitova A., Barsyte-Lovejoy D. et al. Direct interaction between the PRDM3 and PRDM16 tumor suppressors and the NuRD chromatin remodeling complex. Nucleic Acids Res. 2019; 47:1225–1238. 10.1093/nar/gky1192 PMC6379669 30462309

[53] Mohrin M., Chen D. The mitochondrial metabolic checkpoint and aging of hematopoietic stem cells. Curr. Opin. Hematol. 2016; 23:318–324. 26945277 10.1097/MOH.0000000000000244 PMC4891268

[54] Moon J., Kim H.R., Shin M.G. Rejuvenating aged hematopoietic stem cells through improvement of mitochondrial function. Ann. Lab. Med. 2018; 38:395–401. 29797808 10.3343/alm.2018.38.5.395 PMC5973912

[55] Guidez F., Howell L., Isalan M., Cebrat M., Alani R.M., Ivins S., Hormaeche I., McConnell M.J., Pierce S., Cole P.A. et al. Histone acetyltransferase activity of p300 is required for transcriptional repression by the promyelocytic leukemia zinc finger protein. Mol. Cell Biol. 2005; 25:5552–5566. 15964811 10.1128/MCB.25.13.5552-5566.2005 PMC1156991

[56] Vasanthakumar A., Xu D., Lun A.T., Kueh A.J., van Gisbergen K.P., Iannarella N., Li X., Yu L., Wang D., Williams B.R. et al. A non-canonical function of Ezh2 preserves immune homeostasis. EMBO Rep. 2017; 18:619–631. 28223321 10.15252/embr.201643237 PMC5376973

[57] Kasler H.G., Lee I.S., Lim H.W., Verdin E. Histone deacetylase 7 mediates tissue-specific autoimmunity via control of innate effector function in invariant natural killer T cells. Elife. 2018; 7:e32109. 29664401 10.7554/eLife.32109 PMC5943034

