# Supplementary material for: PLZF limits enhancer activity during hematopoietic progenitor aging
Source: Nucleic Acids Res. 2019 Mar 20;47(9):4509–20. doi: 10.1093/nar/gkz174 (PMC6511862; doi:10.1093/nar/gkz174)
Supplement: Supplementary Data [file gkz174_supplemental_files.zip › Revised Poplineau supplemental_Texte+figures.pdf]

SUPPLEMENTAL MATERIALS

Antibodies

Antibodies	Application	Supplier	Reference	Dilution/quantity
SAV PE-CF594	Cytometry	BD Biosciences	562284	1/500
Cd45-2 AF700	Cytometry	BioLegend	109822	1/400
c-Kit APC-Cy7	Cytometry	eBioscience	47-1171-82	1/500
Sca1 PerCP-Cy5-5	Cytometry	BioLegend	122523	1/500
FcgR Pe-Cy7	Cytometry	eBioscience	25-0161-82	1/1000
Cd34 FITC	Cytometry	Miltenyi	120-014-229	1/100
H3K27ac	ChIP	Active Motif	39133	4µl
H3K4me1	ChIP	Abcam	ab8895	1µg
H3K4me3	ChIP	Abcam	ab8580	1µg
H3K27me3	ChIP	Abcam	ab6002	2µg
Flag M2	ChIP ; WB	Sigma	F1804	2µg ; 1/500
PLZF, clone 2A9	WB	Active Motif	39987	1/500
Alpha-Tubulin	WB	Sigma	T6199	1/5000

Primers

Primers ChIP	Forward	Reverse
Hoxd10	5'-GCCTTCCAGAAGACAGGAGC-3'	5'-GGGGTGGCAACTCTGTTCAT-3'
Cd47 enhancer	5'-GGGGAGCCAGTCACCATAGA-3'	5'-GCCTATGCCAGAACGATGGA-3'
Mef2c enhancer	5'-AAGATGGTCTGTGGAGGGGA-3'	5'- AGGAGGCTTGAAAAGCAGCA -3'
Furin enhancer	5'-CCAGTGGTAGCTCAGCTGTT-3'	5'-TTGTTCATGTGTCAGCGTGC-3'

SUPPLEMENTAL EXPERIMENTAL PROCEDURES

Chromatin Immunoprecipitation

Fixed pellets were washed twice with cold PBS and lysed with Lysis Buffer A (0.25% Triton X100, 10 mM Tris-HCl pH8, 10 mM EDTA, 0.5 mM EGTA), and Lysis Buffer B (250 mM NaCl, 50 mM Tris-HCl pH8, 1 mM EDTA, 0.5 mM EGTA). Nuclei were suspended in Buffer C (0.5% SDS, 10 mM Tris-HCl pH8, 1mM EDTA, 0.5 mM EGTA) and chromatin was sonicated using the Bioruptor® Pico (Diagenode) in order to obtain DNA fragments with an average size of 300 bp. After centrifugation, the soluble chromatin was diluted in Buffer D (0.6% Triton X100, 0.06% NaDOC, 150 mM NaCl, 12 mM Tris-HCl pH8, 1 mM EDTA, 0.5 mM EGTA) and immunoprecipitated overnight at 4°C with

magnetic beads (Active Motif) pre-incubated with the antibody of interest (supplemental materials). Buffer A, B, C, D were supplemented with 1X proteinase inhibitor cocktail (Life Technology # 11873580001) and 0.5 mM PMSF. ChIP were washed with the following combinations of wash buffers: W1 (1% Triton X100, 0.1% NaDOC, 150 mM NaCl, 10 mM Tris-HCL pH8), W2 (0.5% NP40, 0.5% Triton X100, 0.5% NaDOC, 150 mM NaCl, 10 mM Tris-HCL pH8), W3 (0.7% Triton X100, 0.1% NaDOC, 250 mM NaCl, 10 mM Tris-HCL pH8), W4 (0.5% NP40, 0.5% NaDOC, 250 mM LiCl, 20mM Tris-HCL pH8, 1mM EDTA, W5 (0.1% NP40, 150 mM NaCl, 20 mM Tris-HCL pH8, 1 mM EDTA), W6 (20 mM Tris-HCL pH8, 1 mM EDTA). Immunoprecipitated DNA from GMPs was purified with I-Pure Kit (Diagenode). DNA from 416b was eluted in Elution Buffer (0.5 mM EDTA, 300 mM NaCl, 0.5% SDS, 10 mM Tris-HCl), reverse cross-linked, treated with RNAse A (Sigma-Aldrich) and Proteinase K (Roche), and purified with a QIAquick PCR purification kit (Qiagen).

For Flag ChIP-seq, 20 millions of cells were fixed with 1% of formaldehyde for 10 min at 25°C. Reaction was quenched by adding 14 volumes of PBS containing 5% of FCS. Fixed pellets were washed twice with cold PBS and lysed in RIPA buffer supplemented with 1X PIC (50 mM Tris-HCL pH8, 150 mM NaCl, 2 mM EDTA, 1% NP40, 0.5% NaDOC, 0.1% SDS). Sonication was done using Ultrasonic Homogenizer and chromatin was incubated overnight at 4°C with magnetic beads (Dynabeads, Invitrogen) pre-incubated with Flag-M2 antibody (supplemental materials). ChIP was washed with the following buffers: twice with WF1 (10 mM Tris-HCl pH8, 200 mM NaCl, 1 mM CaCl2, 0.5% NP40), twice with WF2 (10 mM Tris-HCl pH8, 500 mM NaCl, 1 mM CaCl2, 0.5% NP40), twice with WF3 (50 mM Tris-HCl pH8, 10 mM EDTA). DNA elution was done as described above for 416b cells.

Biological Replicates for ChIP-seq, ATAC-seq and RNA-seq analyses

For 416b cells: histone mark ChIP-seq were done once, but validated by two independent spike-in ChIP-qPCR; Flag ChIP-seq in 416b PLZF-Flag condition were issued from two independent IPs and sequencing, and in 416b Empty-vector condition from one IP and sequencing; ATAC-seq data were issued from two individual experiments; RNA-seq was performed once and data were therefore presented as MA plot.

For GMPs: histone mark ChIP-seqs were issued from two independent experiments (except for H3K4me3) and RNA-seq from three independent RNA extraction and sequencing.

For each model, replicate concurrency was verified using correlation heatmap (Pearson correlation) from Deeptools suite (v2.2.4), and sample replicates were merged into a single bam file.

Computational analyses

Sequencing quality control was determined using FastQC tool (http://www.bioinformatics.babraham.ac.uk/projects/fastqc/). Reads with Phred quality score less than 30 were filtered out.

For ChIP-seq in 416b cells (Flag, H3K4me1, H3K4me3, H3K27ac, H3K27me3), reads were mapped to an "hybrid genome" combining Mus musculus (GRCm38/mm10) and Drosophila melanogaster (dm6) genomes as previously described (1) using default parameters of Bowtie2 (v2.3.4.1); (2). For ChIP-seq in human KG1 cells (PLZF and H3K4me3 from GSE109619, new H3K4me1 and H3K27ac), reads were mapped to Homo Sapiens (hg19) genome. Next, tags were sorted according to their genome of origin (mm10, hg19 or dm6). Duplicate tags were removed and mapped tags were processed for further analysis.

High confidence binding sites were determined using MACS2 peak caller (3): sharp mode for Flag-PLZF (-p 0.05, IDR 1%); broad mode for histone marks (broad-cutoff=0.05 -q 0.01). Inputs were used as controls. For quantitative analysis, mm10 mapped tags were counted within peak coordinates (defined previously by MACS2) using featureCounts (4). For 416b cells, a scaling factor was calculated using dm6 mapped tags (H2Av-bound regions) as described (5). Counted mm10 mapped tags were normalized according to this scaling factor (Spike-in correction). Spike-in correction was done for signal analysis. For IGV visualization, spiked-mapped tags were converted into BigWig using the deepTools suite (v2.2.4) (bamCoverage, multiBigwigSummary) (6).

Active enhancer coordinates were defined by the presence of H3K4me1 and H3K27ac and subsequently depleted for H3K4me3 (7). Furthermore, to determine PLZF-bound enhancers, regions located within 12.5 kb of each other were stitched together as previously described (8). Finally, H3K27ac fold changes were used for enhancer differential binding analysis.

Since a peak is not a single entity and can intersect with multiple peaks, it is worth noting that during intersect processes; we needed to merge peaks for comparison. This can lead to some small variations where, a same dataset can have different total peak number, according to the considered intersection. Peaks were intersected using bedtools intersect (v2.17.0) with a minimum overlap of 1-base (9).

For ATAC-seq, reads were mapped to mm10 and normalized using the number of mouse mapped tags. Peak calling was done using MACS2, sharp mode (-p 0.01). Other analyses were done as for ChIP-seq.

For RNA-seq, reads were mapped to mm10 genome using subread-align (v1.5.0) (10) with default parameters. Gene expression was determined by counting mapped tags at gene levels using featureCounts (4) and counts were normalized by the size of each library (DESeq2, estimateSizeFactors function) (11).

The Gene Ontology (GO) biological processes associated with candidate genes were determined using g:Profiler (12) tool with a p-value < 0.05 and by taking into account a background. For enhancers named "K27ac de novo", "K27ac up", "K27ac unchanged" (supplemental Figure S4) the background used was all active enhancer-associated genes contained in the 416b PLZF-Flag and 416b Empty vector conditions. For enhancers named "common" (Figure 5E), the background used was all active-enhancer-associated genes contained in WT, *Zbtb16*$^{lu/lu}$, Old and Young GMP conditions.

De novo binding motif analysis was performed on GMP active enhancer coordinates. Upon PLZF inactivation, enhancers without H3K27ac variation and enhancers with H3K27ac variation and associated with metabolic processes were analyzed separately using Regulatory Sequence Analysis Tools (RSAT) (13). Only motifs with E-value < 0.05 were filtered-in. Related binding motifs were found using the Catalog of Inferred Sequence Binding Preferences (cis-BP mouse) database (14).

References

1. Orlando, D.A., Chen, M.W., Brown, V.E., Solanki, S., Choi, Y.J., Olson, E.R., Fritz, C.C., Bradner, J.E. and Guenther, M.G. (2014) Quantitative ChIP-Seq normalization reveals global modulation of the epigenome. *Cell Rep*, **9**, 1163-1170.
2. Langmead, B. and Salzberg, S.L. (2012) Fast gapped-read alignment with Bowtie 2. *Nat Methods*, **9**, 357-359.
3. Zhang, Y., Liu, T., Meyer, C.A., Eeckhoute, J., Johnson, D.S., Bernstein, B.E., Nusbaum, C., Myers, R.M., Brown, M., Li, W. *et al.* (2008) Model-based analysis of ChIP-Seq (MACS). *Genome Biol*, **9**, R137.
4. Liao, Y., Smyth, G.K. and Shi, W. (2014) featureCounts: an efficient general purpose program for assigning sequence reads to genomic features. *Bioinformatics*, **30**, 923-930.
5. Egan, B., Yuan, C.C., Craske, M.L., Labhart, P., Guler, G.D., Arnott, D., Maile, T.M., Busby, J., Henry, C., Kelly, T.K. *et al.* (2016) An Alternative Approach to ChIP-Seq Normalization Enables Detection of Genome-Wide Changes in Histone H3 Lysine 27 Trimethylation upon EZH2 Inhibition. *PLoS One*, **11**, e0166438.
6. Ramirez, F., Ryan, D.P., Gruning, B., Bhardwaj, V., Kilpert, F., Richter, A.S., Heyne, S., Dundar, F. and Manke, T. (2016) deepTools2: a next generation web server for deep-sequencing data analysis. *Nucleic Acids Res*, **44**, W160-165.
7. Rada-Iglesias, A., Bajpai, R., Swigut, T., Brugmann, S.A., Flynn, R.A. and Wysocka, J. (2011) A unique chromatin signature uncovers early developmental enhancers in humans. *Nature*, **470**, 279-283.
8. Whyte, W.A., Orlando, D.A., Hnisz, D., Abraham, B.J., Lin, C.Y., Kagey, M.H., Rahl, P.B., Lee, T.I. and Young, R.A. (2013) Master transcription factors and mediator establish super-enhancers at key cell identity genes. *Cell*, **153**, 307-319.
9. Quinlan, A.R. and Hall, I.M. (2010) BEDTools: a flexible suite of utilities for comparing genomic features. *Bioinformatics*, **26**, 841-842.
10. Liao, Y., Smyth, G.K. and Shi, W. (2013) The Subread aligner: fast, accurate and scalable read mapping by seed-and-vote. *Nucleic Acids Res*, **41**, e108.
11. Love, M.I., Huber, W. and Anders, S. (2014) Moderated estimation of fold change and dispersion for RNA-seq data with DESeq2. *Genome Biol*, **15**, 550.
12. Reimand, J., Arak, T., Adler, P., Kolberg, L., Reisberg, S., Peterson, H. and Vilo, J. (2016) g:Profiler-a web server for functional interpretation of gene lists (2016 update). *Nucleic Acids Res*, **44**, W83-89.

13. Thomas-Chollier, M., Herrmann, C., Defrance, M., Sand, O., Thieffry, D. and van Helden, J. (2012) RSAT peak-motifs: motif analysis in full-size ChIP-seq datasets. *Nucleic Acids Res*, **40**, e31.
14. Weirauch, M.T., Yang, A., Albu, M., Cote, A.G., Montenegro-Montero, A., Drewe, P., Najafabadi, H.S., Lambert, S.A., Mann, I., Cook, K. *et al.* (2014) Determination and inference of eukaryotic transcription factor sequence specificity. *Cell*, **158**, 1431-1443.

Figure S1

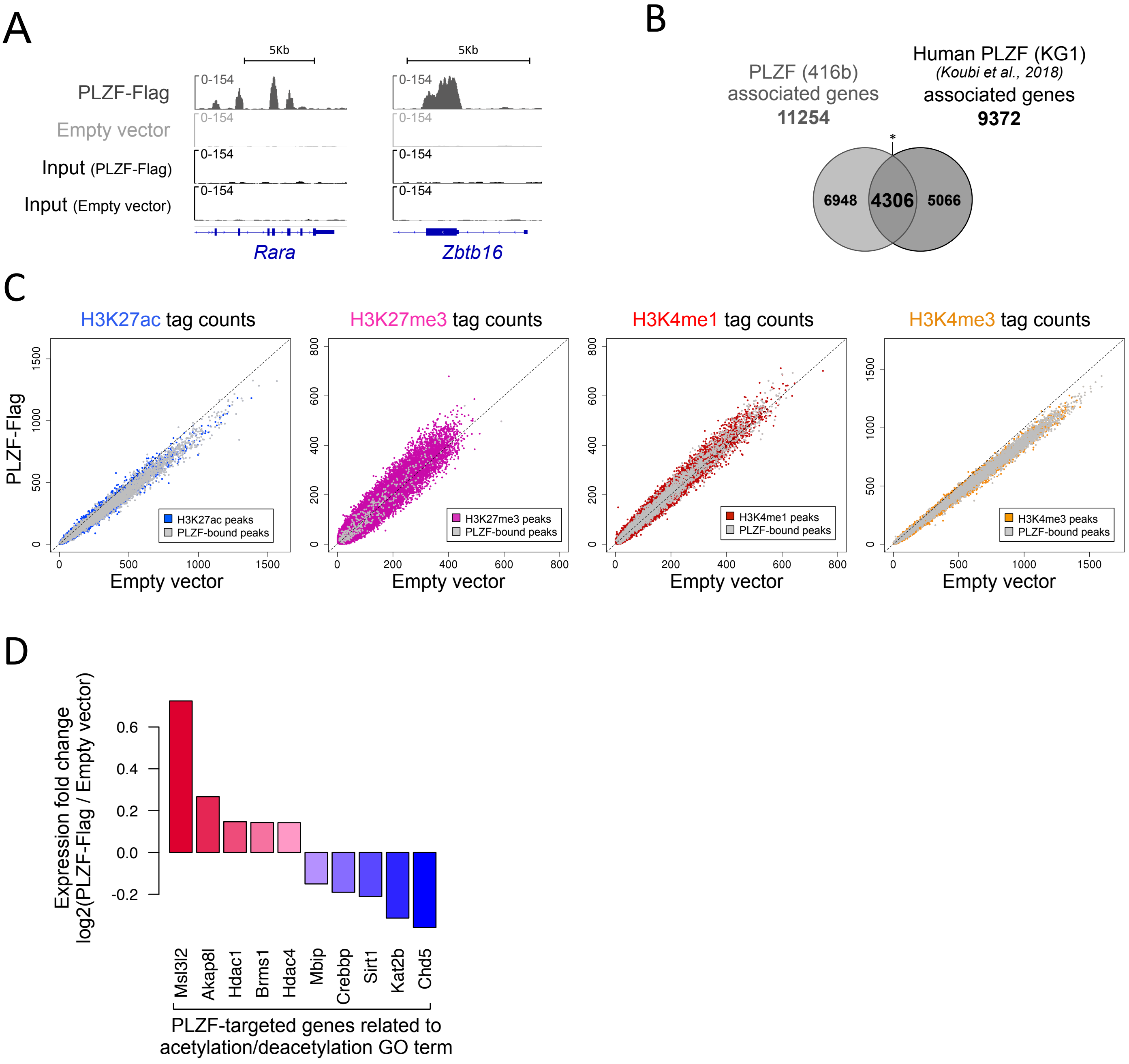

Figure S1. Related to figure 1
(A) Representative Integrative Genomics Viewer (IGV) screenshot of PLZF peaks (PLZF-Flag) on previously described targeted genes. (B) Venn diagrams showing the intersection between PLZF target genes in 416b PLZF-Flag 416b and in human myeloid KG1 cells. PLZF ChIP-seq was extracted from GSE109619. *P < 0.05 (hypergeometric test). (C) Scatter plots showing histone mark profiles in PLZF-Flag *versus* Empty vector conditions. Grey dotes represent sites bound by PLZF. (D) mRNA expression fold change of the 5 more up-regulated and the 5 more down-regulated genes related to acetylation/deacetylation GO terms. Results are expressed as log2(PLZF-Flag/Empty vector) and are extracted from RNA-seq data (n=1).

Figure S2

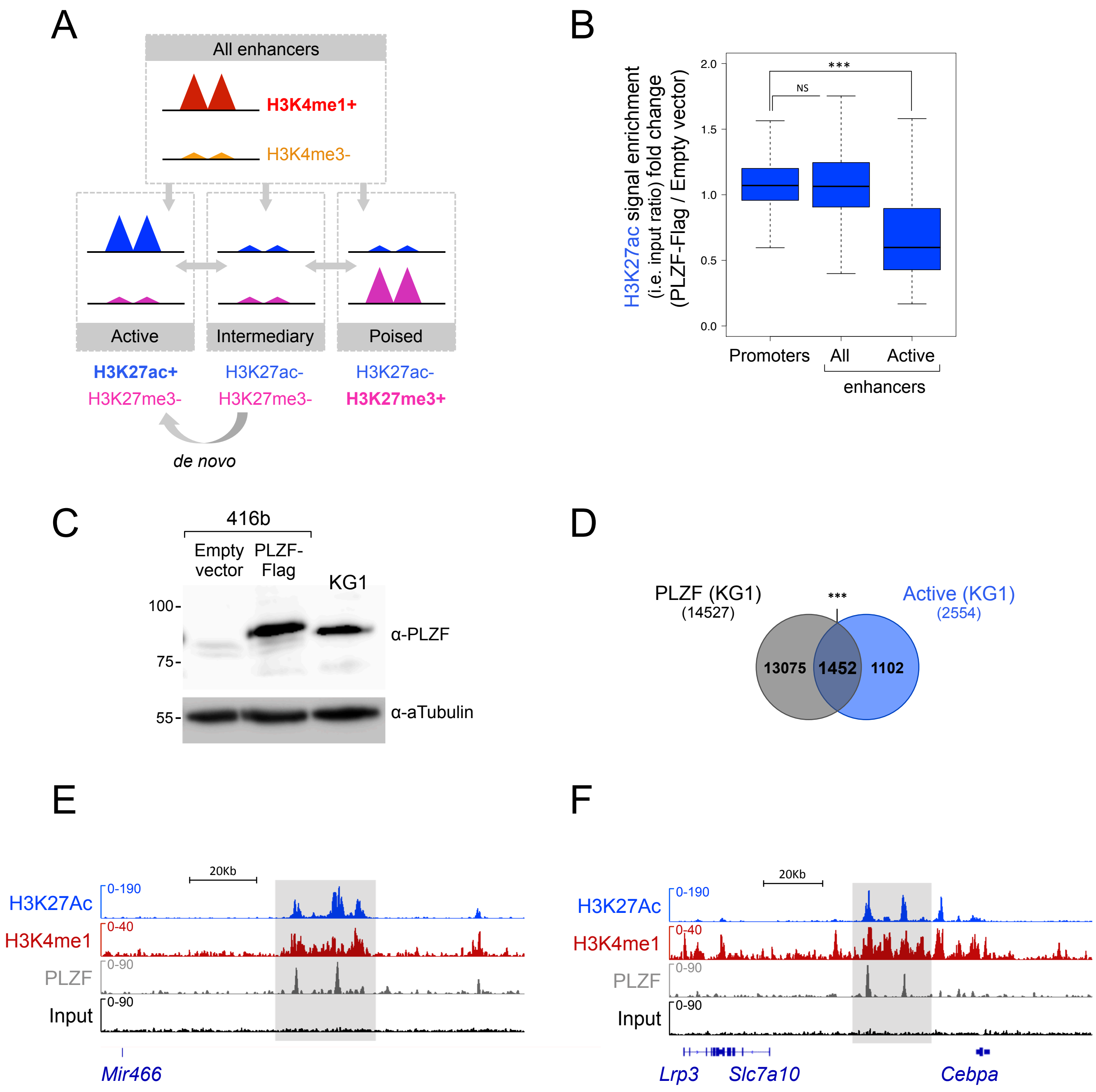

Figure S2. Related to figure 2
(A) Schematic representation of enhancers. Enhancer regions are determined with H3K4me1 (high) and H3K4me3 (low) levels. Intermediary enhancers lack H3K27ac and H3K27me3 but could gain these histone marks. Active enhancers are H3K4me1+, H3K4me3-, H3K27ac+), poised enhancers are H3K4me1+, H3K4me3-, H3K27me3+. Enhancers gaining H3K27ac are defined as "*de novo* active enhancers". (B) Box plot showing H3K27ac enrichment (% H3K27ac bound / Input) fold change (PLZF-Flag / Empty vector) at promoter, enhancer and active enhancer regions. . ****P* < 0.001 (Welch's t.test); NS not significant. (C) Immunoblot showing PLZF expression in Empty vector, PLZF-Flag 416b and in KG1 cells using anti-PLZF antibody. Anti-aTubulin was used as loading control. (D) Venn diagrams showing the overlap between PLZF peaks and active enhancers (Active) in KG1 cells. Enhancer regions in KG1 were determined as for 416b using KG1 ChIP-seq data (GSE109619; new H3K27ac and H3K4me1). ****P* < 0.001 (hypergeometric test) (E-F) Representative Integrative Genomics Viewer (IGV) tracks of H3K27ac, H3K4me1 and PLZF in KG1 cells at enhancer regions. The grey box underlines the region of interest.

Figure S3

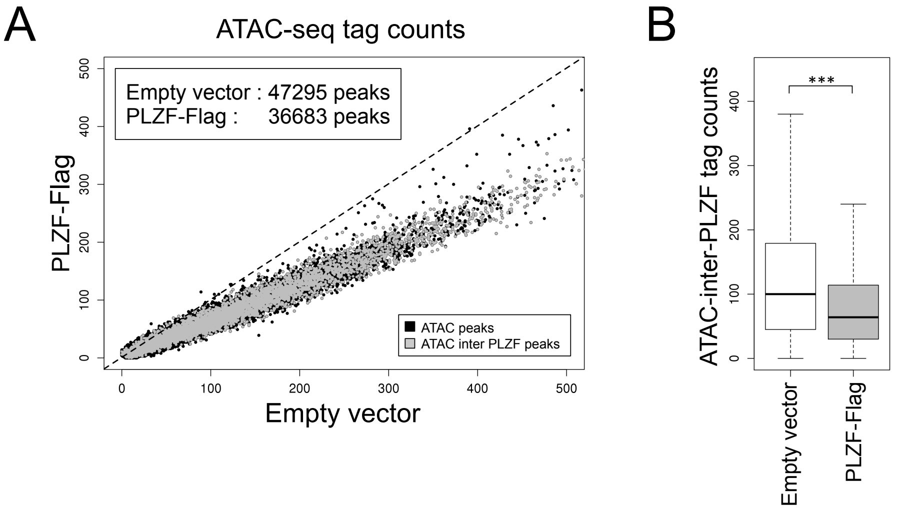

Figure S3. Related to figure 3
(A) Scatter plots showing ATAC-seq signal in PLZF-Flag *versus* Empty vector conditions. Grey circles represent sites bound by PLZF. (B) Box plots showing ATAC-seq signal at PLZF-bound sites in Empty vector and PLZF-Flag conditions. ***$P < 0.001$ (Welch's t.test).

Figure S4

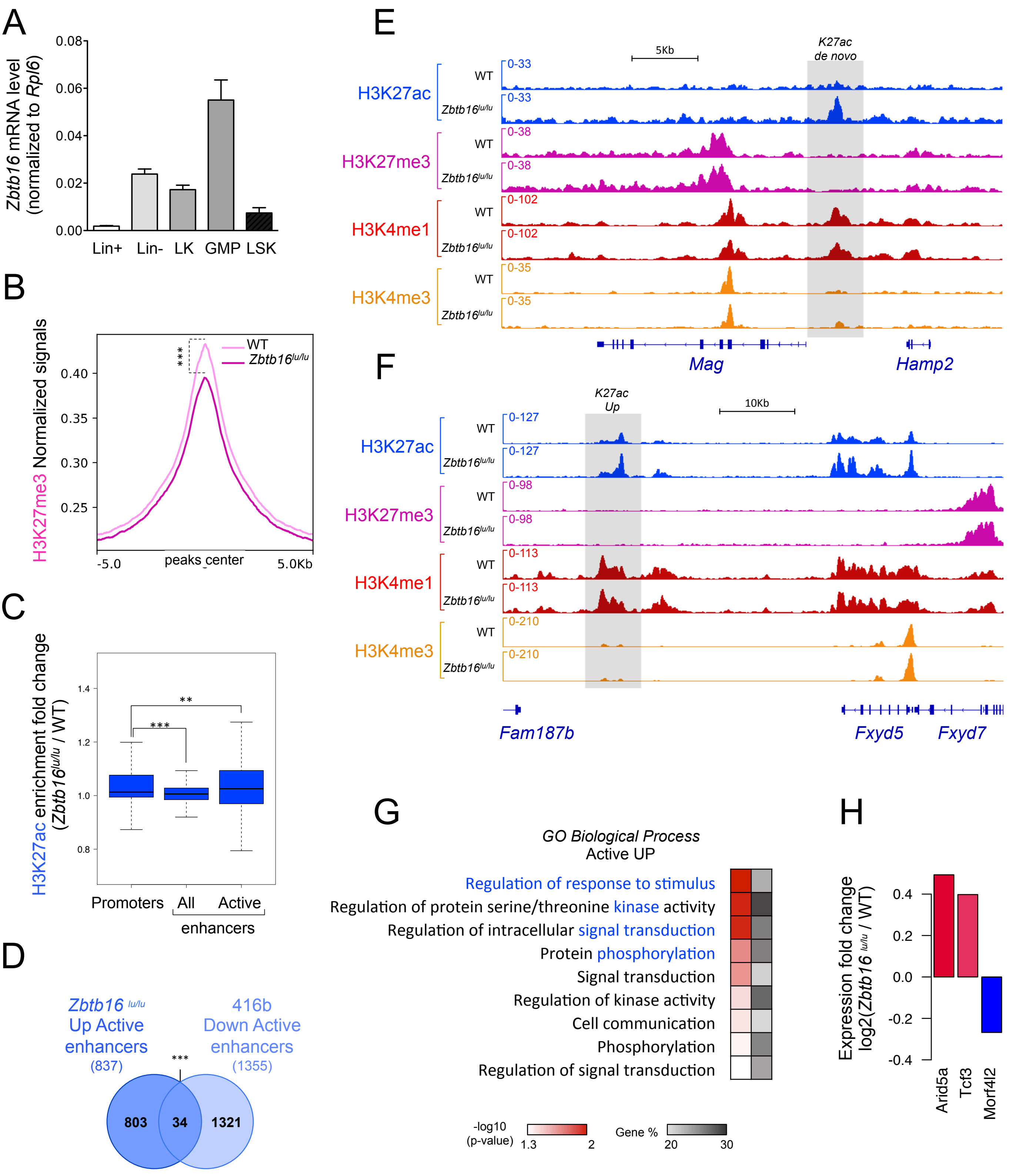

Legend on next page

Figure S4

Figure S4. Related to figure 4

(A) *Zbtb16* expression normalized to *Rpl6* in immature hematopoietic compartment. Lin-: Lineage negative; Lin+: Lineage positive; LK: Lin-, c-Kit+; GMP : Granulocyte-Monocyte Progenitor; LSK: Lin-, c-Kit+ and Sca-1+. mRNA values are expressed as a mean ±SD (n=4). (B) Density plot profiles of H3K27me3 normalized signal in WT and *Zbtb16*$^{lu/lu}$ GMPs . ****P* < 0.001 (paired Welch's t.test). (C) Box plot showing H3K27Ac enrichment (% H3K27ac bound / Input) fold change (*Zbtb16*$^{lu/lu}$ / WT) at promoter, enhancer and active enhancer regions. ***P* < 0.01; ****P* < 0.001 (Welch's t.test). (D) Venn diagrams showing the intersection between enhancers modified upon PLZF over expression (Down Active, from 416b) and upon PLZF mutation (Up Active, from GMPs). ****P* < 0.001 (hypergeometric test) (E-F) Representative Integrative Genomics Viewer (IGV) tracks of H3K27ac, H3K27me3, H3K4me1 and H3K4me3 in WT and *Zbtb16*$^{lu/lu}$ GMPs at enhancer regions. The grey box underlines the region of interest. (G) Gene Ontology (GO) enrichment of genes associated with enhancers that increase (Active UP: K27ac *de novo* and K27ac Up). Red scale indicates the p-value (-log10) and grey scale represents gene % (*i.e.* % of genes observed /total number of genes within each GO term). (H) mRNA expression fold change of acetylation machinery partners. Results are expressed as log2(*Zbtb16*$^{lu/lu}$ /WT) and are extracted from RNA-seq data. *P* < 0.05 (Welch's t.test).

Figure S5

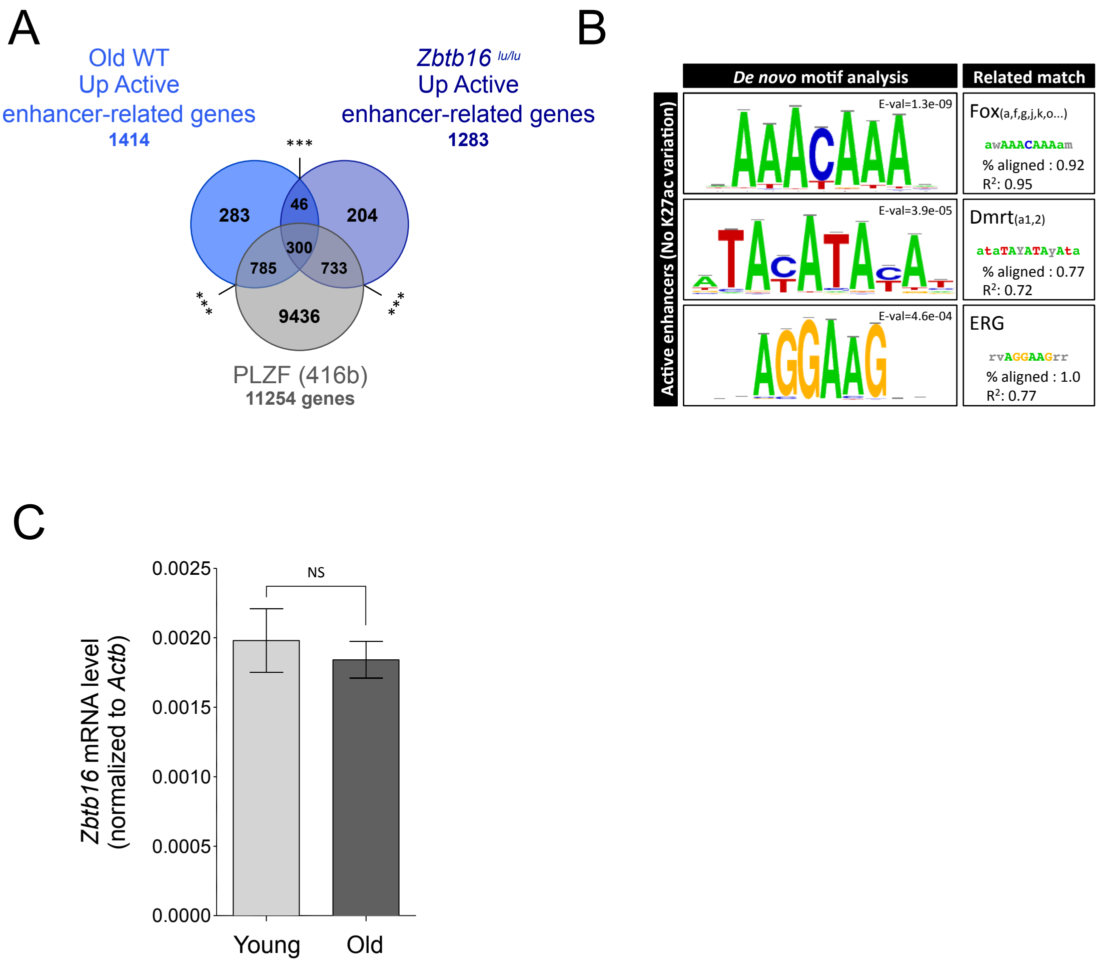

Figure S5. Related to figure 5

(A) Venn diagrams showing the intersection between enhancer-associated genes modified upon aging (Old WT Up Active), modified upon PLZF mutation (*Zbtb16$^{lu/lu}$* Up Active), and PLZF–bound associated genes from PLZF-Flag 416b cells. ***P < 0.001 (hypergeometric test). (B) *De novo* binding motif analysis at active enhancers which were not affected upon PLZF inactivation. (C) RT-qPCR showing *Zbtb16* expression levels in Young and Old GMPs. mRNA values are normalized to *Actb* and expressed as a mean ±SD (n=3); NS not significant.
